# Tapeworm

**Published:** 1860-12

**Authors:** 


### SELECTIONS.

*Tapeworm.*—Dr. Cubbold read before the Middlesex Hospital Medical Society, October 15, a paper on tapeworm, its prevention and treatment. The very interesting observations of recent naturalists upon the development of tapeworm, and their relationship to the cystic entozoa were pointed out, and illustrated by diagrams and specimens. The author then remarked, that to harbor parasitic beings appears to be an almost universal and normal condition of existence. He had himself dissected upwards of six hundred animals belonging to the different vertebrate classes, and had in almost every instance found some form of internal parasite, often many different species, and innumerable individuals inhabiting the same creature. Upwards of twenty species of entozoa are known to infest the human body; of these, four belong to the Tæniadæ or Tapeworm family, viz., Tænia solium, T. mediocanulata, T. nana, and Bothriocephalus latus. The means of prevention are to avoid the introduction of the creature in its undeveloped, or cystic condition into the system. In this state it has received the name of Cysticercus Cellulosæ, and exists frequently in the muscular tissue of the pig, producing what is commonly known as "measly pork," and which, if eaten in an imperfectly cooked state, will infallibly give rise to tapeworm. The treatment recommended was half a drachm of æthereal oil of male fern, mixed with an ounce of honey, half to be taken at night fasting, the other half the next morning, followed in two hours by a brisk purgative.—*Med. Times and Gaz.*, Oct. 27, 1860.

